# Supplementary material for: Why does it take so long for rare disease patients to get an accurate diagnosis?—A qualitative investigation of patient experiences of hereditary angioedema
Source: PLoS One. 2022 Mar 18;17(3):e0265847. doi: 10.1371/journal.pone.0265847 (PMC8932585; doi:10.1371/journal.pone.0265847)
Supplement: S1 File — (PDF) [file pone.0265847.s001.pdf]

Interview guide

<u>Questions</u>

1. Please tell us about your experience before obtaining the diagnosis of hereditary angioedema (HAE).
   - What symptoms did you have, and how did you address them (e.g., visit a medical institution)?
   - Regarding any visits to a hospital, please tell us the following details (as much as you can remember).
     - symptoms which were complained of
     - reason or purpose for consultation
     - type of medical institution, the department within the institution, and how they were chosen
     - content of consultation and examination
     - alternative diagnosis
     - content of treatment, response of medical personnel, etc.
   - If you experienced any interruptions to your life, please describe it.
     - Interruptions to daily life
     - psychological impact
     - interpersonal relationships, etc.
   - How many medical institutions did you consult between the first onset of symptoms and the final diagnosis?
   - Apart from seeking medical attention, have you taken any other action to try to improve your symptoms or other interruptions to your life?

2. How have these problems changed from the time you were undiagnosed to now that you have a confirmed diagnosis?
   - If you have any ideas on how to improve or work on the problems you have faced, please let us know.

3. How did your perception of the disease (symptoms) and your feelings toward medical care (medical professionals and hospitals) change from the time when you were undiagnosed to the present?
   - Currently, how do you view the undiagnosed period?
